# Review of Three-Dimensional Liquid Chromatography Platforms for Bottom-Up Proteomics

**DOI:** 10.3390/ijms21041524

**Published:** 2020-02-23

**Authors:** Van-An Duong, Jong-Moon Park, Hookeun Lee

**Affiliations:** College of Pharmacy, Gachon University, Incheon 21936, Korea; anduong@gachon.ac.kr (V.-A.D.); bio4647@naver.com (J.-M.P.)

**Keywords:** proteomics, 3D separation, mass spectrometry, offline 3D-LC, online 3D-LC

## Abstract

Proteomics is a large-scale study of proteins, aiming at the description and characterization of all expressed proteins in biological systems. The expressed proteins are typically highly complex and large in abundance range. To fulfill high accuracy and sensitivity of proteome analysis, the hybrid platforms of multidimensional (MD) separations and mass spectrometry have provided the most powerful solution. Multidimensional separations provide enhanced peak capacity and reduce sample complexity, which enables mass spectrometry to analyze more proteins with high sensitivity. Although two-dimensional (2D) separations have been widely used since the early period of proteomics, three-dimensional (3D) separation was barely used by low reproducibility of separation, increased analysis time in mass spectrometry. With developments of novel microscale techniques such as nano-UPLC and improvements of mass spectrometry, the 3D separation becomes a reliable and practical selection. This review summarizes existing offline and online 3D-LC platforms developed for proteomics and their applications. In detail, setups and implementation of those systems as well as their advances are outlined. The performance of those platforms is also discussed and compared with the state-of-the-art 2D-LC. In addition, we provide some perspectives on the future developments and applications of 3D-LC in proteomics.

## 1. Introduction

Proteomics is the study of proteomes, aiming at globally characterizing all proteins in any given cell and evaluating protein functions, modifications, and interactions between them [1]. As proteins play a crucial role in cell structure, metabolic processes, and regulatory mechanisms, proteomics is beneficial to assess current cell status, and thereby elucidate disease mechanisms and identify potential candidates for biomarker [2,3,4,5]. However, proteomes are much more complex and dynamic than genomes, as evidenced by the presence of a larger number of proteins compared to genes, which raises more challenges to proteomics from separation to detection [6]. Proteome separation and identification are mainly based on two analytical strategies: Top-down and bottom-up approaches. Top-down proteomic studies use intact proteins for direct separation and identification, usually with liquid chromatography-tandem mass spectrometry (LC-MS/MS) [7]. Top-down proteomics has unique advantages for the comprehensive analysis of proteoforms, which arise from genetic variations, alternative RNA splicing, and post-translational modifications (PTMs) [8]. However, it still faces a number of challenges related to protein’s solubility, separation, ionization efficiency in MS analysis and quantification [9]. In contrast, the bottom-up proteomic approaches minimize the problems by analyzing peptides prepared from enzymatic proteolysis. However, the increased sample complexity requires higher separation power. To this end, multidimensional (MD) chromatography increasing peak capacity dramatically becomes the essential tool in proteome-wide analysis [10,11,12,13].

The simultaneous separation of a large number of peptides (up to hundred-thousands or millions) in a bottom-up proteomic sample raises great challenges to both LC and MS/MS. The depth of proteome coverage has been driven by developments in these techniques. The mass spectrometer has been considerably improved in the past decade with a dramatic increase in acquisition speed and mass accuracy as well as the introduction of new fragmentation techniques [14,15,16,17]. Regarding LC, various MD separation approaches have been developed to decrease the complexity of samples, by which, peptides are consecutively separated by two or more independent separation mechanisms [6,18,19]. MD-LC systems can be carried out in an online or offline manner. Ideally, each dimension in an MD-LC system is completely orthogonal to the others, and the theoretical peak capacity is defined as the product of the individual peak capacity. However, currently available MD-LC systems do not reach the perfect orthogonality due to their intrinsic imperfection and some practical limitations (e.g., band broadening caused by dead volumes) [6,20,21]. After the development of the first two-dimensional LC (2D-LC) system, which was multidimensional protein identification technology (MudPIT) [22], various combinations of MD-LC have been reported. Most of them are 2D-LC with various combinations, such as strong cation exchange chromatography (SCX)-reversed phase liquid chromatography (RPLC) [23,24,25,26,27,28], strong anion exchange chromatography (SAX)-RPLC [29], size exclusion chromatography (SEC)-RPLC [30,31], hydrophilic interaction chromatography (HILIC)-RPLC [32,33,34], RPLC-capillary zone electrophoresis (CZE) [35], and RPLC-RPLC [36,37,38,39,40]. 

Some previous reviews summarized the developments and advances of MD-LC systems and their applications in proteomics, most of which focused on 2D-LC systems [6,18,19,41,42,43,44,45,46,47]. In this review, we summarized recent advances in the development of 3D-LC separation techniques for proteomic application. Existing 3D-LC systems were classified and discussed regarding their setups. The power of 3D-LC compared to 2D-LC in increasing sensitivity and throughput for qualitative analysis is also discussed. Finally, we presented some perspectives on the future developments and applications of 3D-LC in proteomics.

## 2. Recent Developments and Applications of 3D-LC

Generally, an MD separation can be carried out in an online or offline manner. An offline setup is based on fractions’ collection in the first (and second) dimension and their analysis in the final dimension fashion. Each separation process of an offline system is conducted separately, suitable for conventional LC-MS/MS systems. Conversely, in an online platform, the eluent is directly transferred from the first dimension onto the next ones without flow interruption. Online systems, therefore, require unique and sophisticated setups [18]. In this review, existing 3D-LC platforms are classified into offline, online, and combined systems.

### 2.1. Offline 3D-LC Systems

At the early stage of 3D-LC, several 3D-LC systems were developed by modification of MudPIT. Most of them were based on the combination of SCX and RPLC since SCX-RPLC showed high orthogonality and high resolving power [48,49,50]. SCX enables peptide separation with respect to their ionic properties [51,52], whereas RPLC separates them based on their hydrophobicity to long carbon chain resins [53]. Utilizing this basis, Hood et al. performed 3D-LC (weak anion exchange/weak cation exchange-SCX-RPLC) to identify 1057 protein groups from mouse serum [54]. Later, Zhang et al. developed SEC-SCX-RPLC for proteomic analysis of normal human liver tissue with 1622 identified proteins [55]. Besides the development of SCX-RPLC, the combination of two RPLC implemented under extremely different pH conditions (usually high pH and low pH) was proven to be effective [56]. In 2006, Shen et al. described a 3D-LC system (RP-SCX-RP) for investigation of plasma proteome of patients with sepsis and systemic inflammatory response syndrome, which found 484 proteins with gene identification. This 3D-LC platform combined the two most popular 2D-LC approaches, SCX-RPLC and RPLC-RPLC [57]. After that, various offline 3D-LC were developed for proteomic applications, most of which utilized either SCX-RPLC or RPLC-RPLC.

Betancourt et al. developed a workflow consisting of SCX-RP-RP for proteomic profiling of mouse embryonic fibroblasts. Peptides were first subjected to the reversible labeling of their NH_2_ groups. Subsequently, they were separated according to their charge state by SCX into three fractions: neutral, singly, and multi-charged peptides. The peptides were further fractionated using RPLC at pH 10 to generate 30 SCX-RP fractions prior to RPLC (pH 2)–MS/MS analysis. This 3D-LC system allowed the identification of 5051 proteins from 29,843 peptides. This approach required the acylation of peptides’ amino groups before SCX fractionation and the regeneration of amino groups after that [58]. Zhao et al. utilized the same 3D-LC setup to investigate the human embryonic stem cell proteome. Four subcellular fractions were subjected to SCX and then RPLC fractionation with low-pH solvents (0.1% trifluoroacetic acid). The third separation step was also low-pH RPLC (0.1% formic acid). In total, triplicate analysis of 100 SCX-RPLC fractions resulted in the identification of 3184 proteins, more than 24,000 unique peptides, approximately 500 phosphorylation sites, and 68 sites of O-linked β-N-acetyl glucosamine modification [59]. Compared to the above-mentioned study with a similar setup [58], this study was quite modest regarding the numbers of protein and peptide identification. One possible explanation might come from the use of RPLC solvents. Generally, 2 RPLC dimensions are performed under different pH conditions to achieve good orthogonality [56]. The use of low-pH solvents in both second and third RPLC separation in this study might not demonstrate the best performance of the 3D-LC system. 

Recently, a 3D-LC method was developed for an in-depth human urinary proteomic analysis. Peptides were first fractionated by gel-eluted liquid fraction entrapment electrophoresis (GELFrEE) or liquid-phase isoelectric focusing (LPIEF), which generated 12 and 6 fractions, respectively. Each fraction was further fractionated to 20 sub-fractions by high-pH RPLC prior to low-pH RPLC-MS/MS analysis. The number of protein identification in this study was relatively modest (more than 6000), particularly when considering the high number of fractions (360) and the long analysis time (720 h) [60]. Apart from the above-mentioned combinations, Spicer et al. developed an offline 3D platform consisting of three consecutive RPLC: a low-pH RPLC with heptafluorobutyric acid as an ion-pairing modifier, a high-pH RPLC, and a low-pH RPLC with formic acid as another ion-pairing modifier. A high degree of orthogonality was demonstrated using this 3D-LC system, which could identify over 14,000 proteins with 126 fractions. This 3D strategy required a long analysis time (nearly 8 days) and a relatively high sample amount (720 μg of peptides) [61]. Other combinations arising from either SCX-RPLC or RPLC-RPLC are also available. In a recent study, Boichenko et al. compared five different 2D-LC setups for the analysis of human plasma digests and selected the best two combinations to construct an optimal 3D-LC system: electrostatic repulsion hydrophilic interaction chromatography (ERLIC)-RP (pH 10)-RP (pH 2) [62]. Loroch et al. previously reported a 3D-LC system consisting of ERLIC–ERLIC-RP for the simultaneous analysis of proteome and phosphoproteome from one sample [63]. IEF-SCX-RP was also combined for analysis of human saliva proteome [64] and human plasma proteome [65]. 

HILIC separation was first introduced in 1990 [66] and has become popular over the last few years. It is appropriate to analyze polar compounds and has good complementary selectivity to RPLC. Furthermore, HILIC mobile phase is excellently compatible with that of RPLC, making it suitable to develop HILIC-RPLC 2D-LC systems with high orthogonality [44]. The success of some HILIC-RPLC 2D-LC platforms [32,33,34] resulted in their further utilization in 3D strategies. An SCX-HILIC-RP system was developed for phosphoproteome analysis in human cancer cells (HeLa and K562). The authors combined the strength of Ti^4+^-IMAC and SCX-HILIC separation to maximize the in-depth characterization of the cellular phosphoproteome. The 3D strategy showed beneficial when the sample amount was above 3 mg. It could identify 22,148 unique phosphopeptides and 4708 unique phosphoproteins in K562 cells. In HeLa cells, starting from 500 µg of samples, 11,980 unique phosphopeptides and 3424 phosphoproteins were identified by the 3D-LC system. Obviously, to achieve a high number of phosphopeptides and phosphoproteins in K562 cells, high sample consumption and a long analysis time were required [67]. Another combination, SEC-HILIC-RP, was used for human serum proteome analysis in patients with benign prostate hyperplasia. Different from other bottom-up studies, serum proteins were firstly pre-fractionated using SEC. After that, the proteins were subjected to dialysis exchange and in-solution digestion. The obtained peptides were then further fractionated using zwitterion–ion HILIC (ZIC-HILIC) prior to RPLC-MS/MS analysis [31]. A similar setup was used for proteomic analysis of *Paracoccus denitrificans*. With iTRAQ labeling, the total number of fractions was 32, and the number of protein identification was 2627, covering 52.3% of the putative proteome of the bacteria [68]. Setups and performance of some outstanding 3D-LC systems are summarized in Table 1.

**Figure 1:**
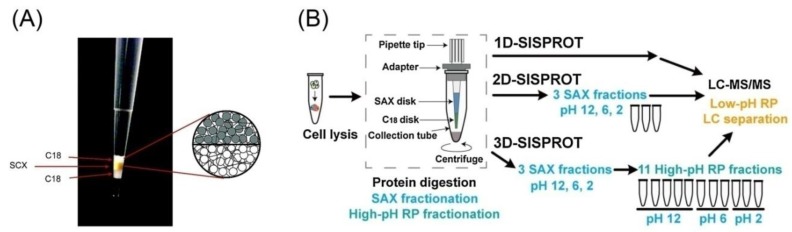
Integrated devices enabling both sample preparation and fractionation. (**A**) Example of a triple StageTip, consisting of C18−SCX−C18-material; reprinted with permission from [71], Copyright (2006) American Chemical Society. (**B**) Three-dimensional (3D)-SISPROT that allows strong anion exchange chromatography (SAX) fractionation followed by high-pH reverse phase liquid chromatography (RPLC) fractionation prior to low-pH RPLC-MS/MS analysis; reprinted and modified from [69], Copyright (2017) with permission from Elsevier.

Generally, offline 3D-LC systems suffer from sample loss due to nonspecific adsorption of samples to tube or other surfaces as well as the additional sample handling. They usually require a large amount of samples in the peptide level [69]. Therefore, a number of studies have integrated the fractionation step into the sample preparation procedure in the same device. These integrated devices could minimize the sample loss, enable a small amount of sample, and ensure high efficient digestion and fractionation. In 2006–2007, stop and go extraction tips (StageTips) consisting of stacking RP and SCX disks were developed for multidimensional fractionation, desalting, filtration, and concentration of peptides [71,72]. Recently, using this device, Kulak et al. reported a simple in-StageTip (iST) method that could enhance the throughput of peptide fractionation and identified 9667 proteins in HeLa cells [73]. Following, Adahi et al. identified more than 22,000 phosphopeptides using this StageTip [74]. As shown in Figure 1A, the StageTips device is an enclosed tip chamber with a membrane working as a filter and separation unit. In this device, SCX fractionation and C18 clean-up were combined. Obviously, these studies performed 2D-LC separation for proteomic analysis, not 3D-LC. However, this design can allow SCX-RP peptide fractionation by using appropriate eluents prior to PRLC-MS/MS analysis, making it a 3D-LC method. With a similar idea, Chen et al. previously developed a simple and integrated spintip-based protein digestion and 3D peptide fractionation technology (3D-SISPROT) with SAX and C18 membranes packed into one pipet tip. These systems allowed all the sample preparation steps to occur consecutively, including protein pre-concentration, reduction, alkylation, digestion, SAX-based fractionation, desalting, and high-pH RP fractionation. The third dimension was low-pH RPLC coupled with MS/MS analysis. 3D-SISPROT could readily identify more than 8000 proteins from 30 µg of cell lysates. As shown in Figure 1B, 3D-SISPROT firstly allowed SAX fractionation of the digested peptides using buffer solutions of different pH (pH 12, 6, and 2, respectively). The peptides from each SAX fraction were then captured by the C18 disk. They were fractionated using high-pH buffer solutions (5 mM ammonium formate (pH 10)) with a stepwise increasing gradient of ACN (3%, 6%, 9%, 15%, and 80% (v/v)). The obtained peptide samples were dried and reconstituted for the low-pH RPLC–MS/MS analysis [69]. Subsequently, this group further developed mixed-mode-SISPROT, in which, SCX and SAX beads were mixed with 1:1 ratio instead of the use of only SAX beads in 3D-SISPROT. In this design, protein digest was fractionated with SCX/SAX mixed-mode and then with high-pH RPLC prior to low-pH RPLC-MS/MS analysis. From 1 mL of serum, 862 proteins were identified with 12 fractions in 12 h of MS/MS analysis [70].

### 2.2. Online 3D-LC Systems

Some online 3D-LC systems have been developed with an attempt to reduce sample loss, labor intensity, and time. They are relatively sophisticated in instruments and typically limited in total sample capacity. In the early stage, after developing MudPIT as the first 2D-LC system, Yates’ group continued to design a 3-phase MudPIT column packed with RP-SCX-RP. It could be considered the first 3D-LC setup for proteomic analysis. However, during implementation, the first RP dimension served as a desalting step, and elution steps were carried out for a 2D separation (SCX-RP) [75]. Similarly, in 2005, another group constructed an RP-SCX-RP column for sample separation to discover yeast proteome [76]. However, with this column, a three-cycle method was carried out, including an RP gradient over 120 min, a salt step of 10 min, and an RP gradient of 120 min corresponding to RP1, SCX, and RP2 separation, respectively. When applying for proteomic analysis of *Saccharomyces cerevisiae*, this 3D-LC platform identified 3019 proteins, in which, approximately 1900 proteins consisted of at least 2 unique peptides [76]. Although no special setup was performed on LC separation, it was a factually online 3D-LC platform without offline pre-fractionation step. Wang et al. also developed an online 3D-LC platform with an RP-SCX biphasic column connected to an RP column prior to MS/MS analysis. This group, however, did not perform the first RP fractionation by adjusting eluents and the system was a 2D-LC platform rather than 3D-LC. Therefore, this system only quantified about 1000 proteins from 30 μg of human liver using 11 SCX fractions [77]. This system was later applied by Song et al. for phosphoproteome analysis; however, the implementation was still 2D-LC separation [78]. Until 2014, based on this setup, Xu et al. developed a combined offline and online 3D-LC system for proteome quantification of hepatocellular carcinoma tissues, which will be discussed in the following section [79].

**Figure 2:**
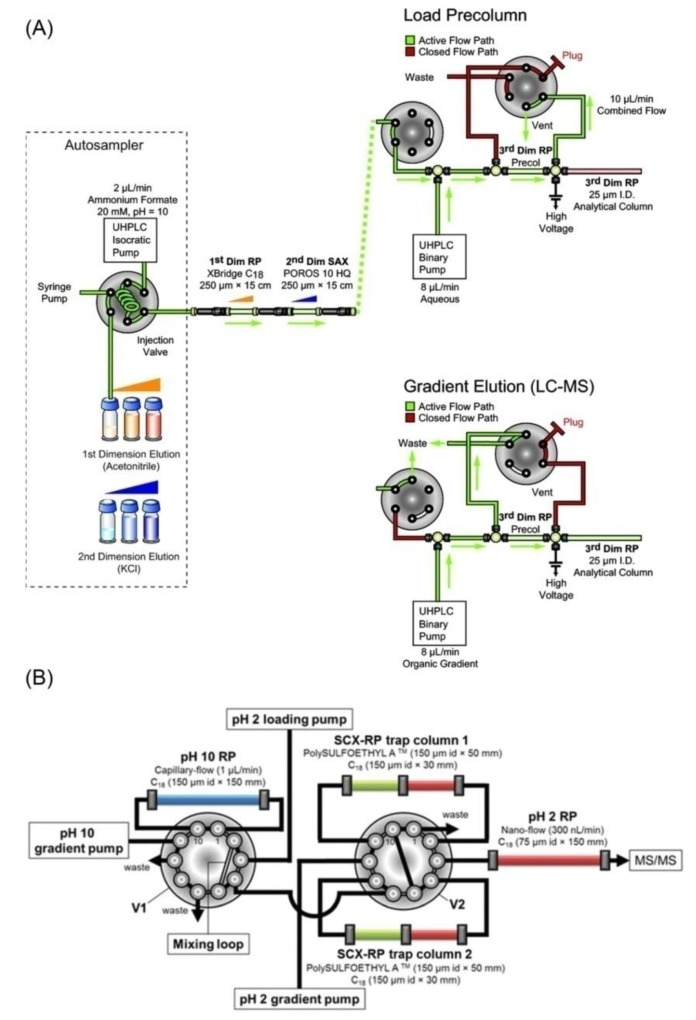
Arrangement in two online 3D-LC platforms. (**A**) Schematic diagram of automated, online nanoflow RP-SAX-RP platform. Reprinted with permission from [80], Copyright (2011) American Chemical Society. (**B**) The detailed layout of the capillary-/nano-flow setup using two switching valves. Reprinted with permission from [83], Copyright (2015) Royal Society of Chemistry.

Besides packing 3-phasic columns, some groups have developed online 3D-LC platforms by modifying setups of HPLC/UPLC systems. In 2011, one group reported an online 3D-LC platform consisting of RP (pH 10)-SAX-RP (pH 2) [80]. In this system, a SAX column was connected directly to the outlet of the first dimension RP column (Figure 2A). The third dimension column was set up using an additional six-port, two-position valve, which ensured the efficient capture of peptides. Online solvent adjustment was performed using 2 pumps. The first pump delivered different discrete eluents (with various ACN and KCl concentration), whereas the second binary pump eluted peptides from the third RPLC dimension column for MS/MS analysis. The system could generate from 19 up to 236 fractions for RPLC-MS/MS analysis depending on the steps in acetonitrile (first dimension) and salt concentrations (second dimension). The optimal achievement was the case of 101 fractions, in which the system identified 4004 proteins and 26,468 peptides from only 5 μg of yeast tryptic peptides. However, when the number of fractions was decreased to 51, there were negligible changes in the protein and peptide identification (only 5% reduction). The online fractionation strategies in this study provided large-scale proteome sequence coverage from only a few micrograms of tryptic peptides. However, the identification of approximately 4000 proteins was not dramatically high compared with recent studies when considering a total of 101 fractions and an analysis time of 202 h [80]. In another study, this group applied the same RP–SAX–RP approach to identify 12,739 phosphopeptides in 126.5 h with a peak capacity of over 3500 [81]. Continuously, by applying the same methodology, the authors developed DEep Efficient Peptide SEquencing and Quantification (DEEP SEQ) mass spectrometry platform to provide increased separation capacity, rapid sequencing speed and quantification of proteins in murine embryonic stem cells. From 20 RP-SAX fractions and combined with iTRAQ-labeling, DEEP SEQ could quantify 211,535 unique peptide sequences that mapped unambiguously to 11,352 proteins. The protein number spanned approximately 70% of the highly curated Swiss-Prot database, indicating the depth and scale of proteome coverage. The major drawback of this study was the long MS time of about 24 days (for triplicate analysis of 20 fractions, each fraction about 9.7 h) [82].

In 2015, another online 3D-LC system consisting of RP (pH 10)-SCX-RP (pH 2) was developed. Three different pumps were used to deliver eluents, and column flow switching was carried out using two ten-port switching valves (Figure 2B). The first pump (pH 10 gradient pump) separated peptides to different high-pH RPLC fractions. Each fraction from the first dimension was collected using a mixing loop and then transferred to one SCX-RP column. The RP portion was used as a trapping column prior to low-pH RPLC-MS/MS analysis. The second pump (pH 2 loading pump) introduced a salt pulse (low or high) to elute peptides from the SCX portion to the RP portion. The peptides were then eluted with a low-pH gradient solvent delivered by the third pump (pH 2 gradient pump). Two SCX-RP columns were used, allowing the simultaneous performance of pumps 2 and 3 as follows: analysis of RP1/SCX1 fraction and elution of RP2/SCX1 fraction, analysis of RP2/SCX1 fraction and elution of RP1/SCX2 fraction, analysis of RP1/SCX2 fraction and elution of RP2/SCX2 fraction, analysis of RP2/SCX2 fraction and elution of RP3/SCX1 fraction. It continued until the last fraction was analyzed. In rat pheochromocytoma PC12 cell, this online 3D-LC system could identify 6345 proteins and 97,309 unique peptides with 12 fractions in 24 h. This platform was amenable to high-throughput analyses with almost no idle time in sample fractionation, trapping, and desalting [83]. 

Overall, online 3D-LC systems for proteomic applications have been developed for many years. At the early stage, packing 2- and 3-phasic columns were mostly used [75,76,77,78]. Later, more sophisticated systems were designed to enhance their performance [80,81,82,83]. Some platforms were proven to be powerful for in-depth proteome analyses despite the fact that they were hard to be set up without well-trained skills. The performance of some online 3D-LC systems is outlined in Table 2. 

### 2.3. Combined Offline-Online 3D-LC Systems

**Figure 3:**
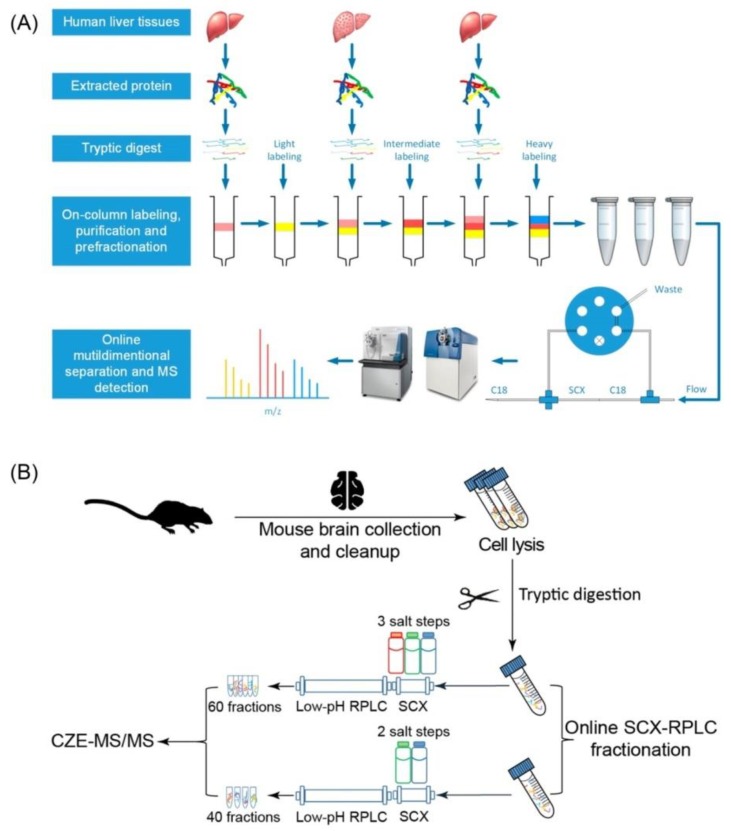
Experimental design of two combined online and offline 3D-LC platforms for proteomic applications. (**A**) Large-scale proteome quantification of human hepatocellular carcinoma and normal liver tissues using high-pH RPLC pre-fractionation and online SCX−RP 2D nanoflow LC−MS/MS analysis. Reprinted with permission from [79], Copyright (2014) American Chemical Society. (**B**) Mouse brain proteome investigation using online SCX-RPLC fractionation and capillary zone electrophoresis (CZE)-MS/MS analysis. Reprinted and modified from [51], Copyright (2018) with permission from Elsevier.

A 3D-LC strategy can be performed using a mixed online and offline system. The sample is either pre-fractionated with the first dimension prior to an online 2D-LC separation or fractionated with an online 2D-LC platform and then separated with the third dimension coupled with MS/MS analysis. Utilizing the former approach, a simple RP-SCX-RP 3D-LC was previously developed by Xu et al. (Figure 3A). This group firstly performed on-column sample purification, stable isotope dimethyl labeling, and high-pH RP pre-fractionation using polystyrene-divinylbenzene beads-packed solid-phase extraction column. Three fractions were collected and subsequently subjected to online SCX–RP 2D-LC-MS/MS analysis [79]. The setup of the online SCX-RP 2D-LC was previously described [77]. Each high-pH RP fraction was loaded onto the RP segment of the RP-SCX biphasic trap column. The peptides were transferred to the SCX segment with a 90 min RP gradient and then to the C18 separation column with a series of 12 stepwise elutions with different salt concentrations at low pH (pH 2.7). After each salt step, a 200 min binary low-pH RP gradient was applied to separate the peptides prior to MS/MS analysis. With 36 RP-SCX fractions and a total MS/MS analysis time of 124 h, this 3D-LC system was successfully applied to identify more than 2700 proteins, access the differential proteome expression of hepatocellular carcinoma tissues and normal liver tissues, and thereby discover dysregulated proteins during the hepatocarcinogenesis process [79]. 

Recently, a different 3D-LC setup was presented, which combined online SCX-RP fractionation and CZE-MS/MS analysis (Figure 3B). The peptides loaded onto the SCX column were eluted with three different concentrations of ammonium acetate (150, 350, and 890 mM). After each salt step, the eluted peptides captured on the RPLC column were further separated with low-pH RPLC gradient solvents to generate 20 fractions. Subsequently, 60 SCX-RPLC fractions were subjected to CZE-MS/MS analysis, which identified about 8200 protein groups and 65,000 unique peptides from a mouse brain proteome digest. This is one of the rare studies that do not use low-pH RPLC as the final fractionation prior to MS/MS analysis [51]. Table 3 summarizes major features of these two combined 3D-LC platforms.

### 2.4. Comparison of Offline and Online Setups

We have discussed the setups and performance of various online, offline, and combined 3D-LC systems, from which, some advantages and limitations of each strategy can be withdrawn. Obviously, offline systems are simple in design and therefore, easy to transfer to other laboratories. Online platforms usually require sophisticated setups and well-trained persons to operate, and therefore they are difficult for technology transfer. Conducting 3D-LC systems in an offline manner can allow a high amount of loading sample. However, this is also one of their drawbacks, since they are not suitable for samples with a low amount, particularly clinical samples. In contrast, online platforms only require a small amount of protein digest (several to 100 μg), making them ideal for some cases when the sample amount is limited [84]. Apparently, if we attempt to increase the numbers of peptide and protein identification through increasing sample amount and fraction number, an offline strategy is the appropriate one, rather than an online strategy. Also, offline fractionation is highly flexible since each dimensional separation can be carried out separately for optimization by varying buffer and elution conditions. In each fractionation process, nonconsecutive fractions can be pooled to reduce the total fraction number depending on the sample complexity [83,84,85]. When the fractionation step is carefully optimized, it can offer critical advantages in terms of the number of fractions reduction and analysis time. One of the major limitations of offline 3D-LC platforms is a high risk of sample loss and contamination, as a result of high-labor intensity with many sample handling steps. The online systems are usually fully automated; samples are continuously delivered between consecutive dimensions through the use of switching valves, additional pumps and trapping columns. The sample loss is thereby considerably low. Online strategies sometimes decrease the overall time from sample preparation to analysis [19,42]. Thus, each strategy has some advantages and drawbacks. Several features in these two strategies comparison are listed in Table 4. Recently, with the development of some integrated devices as has been discussed, offline 3D-LC strategies could overcome their major limitations: sample loss and large sample amount requirement [69,70,71,72,73,74]. These devices, therefore, make the recent offline 3D-LC approaches more potent and applicable to clinical samples. Some combined 3D-LC systems have been developed as discussed above. They might include some advantages and limitations of both offline and online systems. Since there are not many existing combined 3D-LC platforms, it is not easy to compare them with other offline and online systems.

## 3. The Power of 3D-LC Compared to 1D and 2D-LC

Performance comparisons among 3D-LC, 2D-LC, and 1D-LC systems have been reported in some studies, which are summarized in Table 5. In the 3D-SISPROT system, the efficacy of 3D-LC was indicated by comparison of performance among three modes: 1D-, 2D-, and 3D-SISPROT. In 1D-SISPROT, the cell lysates (HEK 293T) were digested and then analyzed by the low-pH RPLC–MS/MS, whereas in 2D-SISPROT, the cell lysates were digested and fractionated on the SAX disk, followed by the low-pH RPLC–MS/MS analysis. The 3D-SISPROT included SAX fractionation, high-pH RPLC fractionation, and low-pH RPLC–MS/MS analysis. As a result, 4275 proteins and 19,768 unique peptides were identified in the 1D-SISPROT mode with only 1.4 h of MS time. Those numbers increased to 5380 proteins and 34,912 unique peptides in the 2D-SISPROT mode with 3 SAX fractions. Taking advantage of the 3D separation strategy, the 3D-SISPROT mode identified 8222 proteins and 74,432 unique peptides with 11 SAX-RP fractions in the MS time of 20.4 h. Thus, 3D-SISPROT intensively increased the numbers of identified proteins and unique peptides in comparison with 2D- and 1D-SISPROT [69]. A previous study also found similar results when comparing the performance of 1D (RP), 2D (RP-RP), and 3D (RP-RP-RP) [61]. Compared with the 2D-LC, the 3D-LC procedure increased the identification of protein and peptide 1.6- and 2.3-fold, respectively. 

Zhou et al. compared the efficacy of 1D, 2D, and 3D systems for the identification of phosphoproteins in HeLa and K562 cells. The author evaluated those strategies in two cases: (i) sample amounts are limited (HeLa cells) and (ii) sample amounts are not limited (K562 cells). In the first case, 3726 unique phosphopeptides originating from 1570 phosphoproteins were identified using 1D-LC. The 2D-LC increased the numbers of phosphopeptides and phosphoproteins to 16,637 and 3891, respectively as HILIC fractionation was utilized. Surprisingly, the overall identifications of phosphopeptides and phosphoproteins in the 3D-LC strategy were 11,980 and 3424, respectively, lower than those obtained from the 2D-LC strategy when using the same starting amount of material. It could be due to the greater total loss of the 3D-LC strategy compared to the 2D-LC strategy. In this case, the sample amount was within the capability of the 2D-LC system; therefore, the use of the 3D-LC was not essential. However, in the second case, when sample amounts were unlimited, the efficacy of the 3D strategy was proven. When the sample amount was increased from 1.5 to 3 mg, the 2D-LC system only provided a 20% increase in the number of phosphopeptides (from approximately 14,000 to nearly 17,000). The dynamic range and intrinsic complexity within each HILIC fraction limited the performance of the 2D-LC system despite the sufficient sensitivity of the final LC−MS step. In the 3D strategy, with 66 SCX-HILIC fractions, the number of phosphopeptides and phosphoproteins in increased considerably to 22,148 and 4708, respectively. Thus, a 3D-LC system is not always superior to a 2D-LC system. When the sample amount is small, the selection of a 2D- or 3D-LC strategy should be carefully considered [67].

In the study of Law et al., the 3D RP–SCX–RP platform outperformed the 2D RP–RP platform in terms of the numbers of identified peptide and protein [83]. When operating the 3D-LC platform, 3114 proteins and 25,792 unique peptides were identified in a single run, higher than those of the 2D-LC platform (2358 proteins and 13,573 unique peptides) and comparable with the data obtained from triplicate runs of the 2D-LC platform (3170 proteins and 23,806 unique peptides). In another study aiming at the identification of human urinary proteins, data obtained from two 3D systems (GELFrEE-RP-RP and LPIEF-RP-RP) were combined. In total, they showed more than 6000 identified proteins, 2-time higher than that of the RP-RP system [60]. A recent study revealed that a 3D-LC (SCX-RP-CZE) produced comparable numbers of protein groups and unique peptides with a state-of-the-art 2D-LC (RP-RP) from the mouse brain proteome digest using comparable MS/MS analysis time (8200 vs. 8900 protein groups, 65,000 vs. 70,000 unique peptides, 70 h vs. 60 h). This study proved for the first time that CZE-MS/MS could approach comparable performance to the cutting-edge 2D-LC-MS/MS for deep proteomic sequencing. Obviously, the 3D-LC did not show superiority compared to the 2D-LC. In this case, the 2D-LC, due to its simple setup and implementation, would be preferred. However, since the separation mechanisms of CZE and RPLC were different, combining those two methods could improve the number of protein groups and unique peptides by about 10% and 40%, respectively, compared with the 2D-LC alone [51]. In the above-mentioned studies, the number of fractions in 3D-LC was always higher than that in 2D-LC. However, a previous study showed that even when the fraction numbers were similar between 2D-LC and 3D-LC, the later still provided more protein and peptide identification. In this study, the online RP-SAX-RP platform could identify 46% and 31% more peptides and proteins, respectively, as compared to the online RP-RP [80]. 

## 4. Author’s Outlook and Concluding Remarks

Significant improvements have been obtained in proteome analysis using 2D-LC. It remarkably provides a higher peak capacity than 1D-LC. However, the resolution might still be limited in the cases of highly complex samples [43]. Therefore, many studies have been conducted to achieve deeper proteome coverage. By adding one more separation stage, some 3D-LC systems have been developed with an attempt to reduce the sample complexity further, increase the peak capacity even more, and improve the efficiency of the LC-MS/MS analysis. The 3D-LC separation requires the selection of three separate dimensions that are highly independent and mutually orthogonal. Also, the mobile phases need to be compatible, particularly with online systems [86]. Although it is a difficult task, various 3D-LC strategies have been developed and widely used. As discussed above, different combinations of LC modes were available in 3D-LC systems. The establishment of a 3D-LC platform depends on some factors, such as experiment type, sample type, and sample amount. Online and offline systems have their own advantages and limitations. Generally, when sample quantity is limiting, one can choose online strategies or use offline platforms with integrated devices. 

It should be noted that the utilization of a 3D-LC system for proteomic analysis is not always the best choice. A 3D-LC system may not be superior to a 2D-LC system when the sample amount is small [67]. In addition, the peak capacity of 3D-LC may not be improved significantly compared to 2D-LC [86]. Sometimes, the selection of three LC modes in 3D-LC does not result in a better performance in comparison with a conventional 2D-LC [51]. In some cases, a 2D-LC is well performed and able to generate satisfactory results. For example, in a recent study, when high-pH RP fractionation and low-pH RPLC-MS/MS analysis were used, approximately 584,000 unique peptides and 14,200 protein isoforms were identified from HeLa cells [87]. Generally, 3D-LC systems suffer from sample loss, an increase in time and sample consumptions. Sample loss is one of the major drawbacks when performing 3D-LC [70]. As a result, 3D-LC methods often require a large amount of sample. It is particularly prominent in offline systems with the use of several hundred micrograms [61] to microgram [68] of peptide sample. However, the development of integrated devices has intensely reduced the sample amount. For example, the 3D-LC method with mixed-mode-SISPROT can be performed with ~ 60 µg of depleted plasma [70]. One can also choose to carry out online 3D-LC for in-depth proteomic analysis in case the sample amount is limited. The sample amount can be reduced to 100–200 µg [76,83] or even less than 100 µg [80,82]. Besides, one of the unavoidable disadvantages of 3D-LC is the long analysis time. As shown in Table 5, the analysis time for 3D-LC is longer than that for 2D-LC at least 3–6 times [61,67,69] or even 18 times [60]. However, there was a study, in which the 3D-LC method required a shorter analysis time compared to the 2D-LC method (74 h vs. 80 h) but still provided higher numbers of protein and peptide identification [80]. Overall, the increase in analysis time for 3D-LC is an inevitable result arising from the increase in the number of fractions. However, depending on each study, we should decide an appropriate fraction number that could be beneficial to the in-depth proteomic data, whereas the analysis time is not so long [51]. In some cases, increasing to a higher fraction number (i.e., longer analysis time) only gains a little increase in peptide and protein identification [80].

In addition, there are two particularly important issues when 3D-LC strategies are applied for label-free quantitation of proteomic samples. The first one is a deterioration of chromatographic profiles for the abundant peptides that may occur due to the increase in peptide load. The second one is an increase in the proportion of unwanted peptide modification due to fractions handling [61]. Another disadvantage with 3D-LC is that a single peak could be split into multiple fractions, making it difficult in a quantitative analysis [6]. For example, when performing DEEP SEQ MS, about 11.5% of all detected peptides span in more than one fraction [82]. It results in difficulties when utilizing label-free quantification in 3D-LC strategies. Moreover, 3D-LC systems may suffer low reproducibility in quantification. However, these issues have been solved in recent studies using different labeling methods. For example, using the pseudo-triplex dimethyl isotope labeling approach, one can obtain two replicated quantification results in just one experiment [78,79]. Loroch et al. used a 2-plex stable isotope labeling by amino acids in cell culture (SILAC) to combine two biological replicates in one experiment and conduct a 3D-LC strategy [63]. Hence, labeling methods can allow the implementation of two or more samples under completely identical conditions, which therefore improves the reproducibility. Other labeling methods such as tandem mass tags (TMT), isobaric tags for relative and absolute quantitation (iTRAQ) can even be used for a quantitative comparison of up to 8 or 10 samples [6]. Thus, the implementation of a 3D-LC strategy usually requires more time and effort and should be compensated by significantly improved output. Furthermore, recent advances in MS have sometimes resulted in satisfactory outcome without MD-LC, such as the implementation of online parallel accumulation–serial fragmentation (PASEF) could identify about 2100 proteins from only 10 ng HeLa digest in 30 min and quantify 5575 protein groups in 2 h LC-MS/MS time across four injections [88]. When the analytical question is to focus on quantitative results, the in-depth proteomic analysis with MD-LC may not be necessary. In this case, one might perform a different strategy, such as sequential window acquisition of all theoretical spectra (SWATH) MS [89] or data-independent acquisition (DIA) MS [90].

Some studies have performed quantitative proteomics with MD-LC strategies to achieve a deep coverage proteome quantification [6,42,44]. It is of great benefit for clinical applications, particularly for the discovery of biomarkers [91]. It was reported that a 3D-LC strategy combined with iTRAQ-labeling could quantify more than 200,000 unique peptides [82]. Ficarro et al. used iTRAQ 4-plex reagent to label peptides from four different samples. After mixing, labeled phosphopeptides were enriched and then subjected to a 3D-LC MS/MS analysis (67 fractions) [81]. Furthermore, in another quantitative proteomic study, 8-plex iTRAQ reagent allowed the parallel analysis of eight samples (4 conditions × 2 replicates) in one experiment [68]. A recent study used 4-plex iTRAQ reagent to label 2 independent biological replicates of peptide samples from a pair of a tumor and an adjacent normal tissue [92]. Using these labeling methods, 3D-LC strategies can enable peptide and protein quantification with high accuracy and reproducibility.

In conclusion, various MD-LC systems have been described so far for peptide separation in proteomic studies. The selection of the most suitable one for an experiment depends on many factors, such as the analytical question, available equipment, sample type, sample amount, available analysis time, and experience as well as skills of the operators. In each case, we have to answer many questions:

– Should we perform an MD-LC method? If yes, is a 2D-LC or a 3D-LC method suitable?
– Which LC modes should be combined?
– If it is a 3D-LC system, should it be offline, online, or combined?

Up to date, the outstanding performance of 3D-LC is undeniable. We believe that 3D-LC systems will be continuously developed, improved, and applied in the future. The development of 3D-LC combined with major improvements in the sensitivity and the resolution of MS/MS analysis will enable increasingly wider proteome identification. Basic 2D-LC combinations such as SCX-RP and RP-RP will be fundamental for future 3D-LC systems. In addition, other LC modes, including HILIC, ERLIC, mixed SCX-SAX will be continuously combined and investigated. The options of 3D-LC strategies will therefore expand. Offline and online 3D-LC systems will be developed simultaneously since they possess their unique advantages. Combined systems may still be developed, taking advantage of the state-of-the-rat 2D-LC strategies. Finally, integrated devices that enable many steps in sample preparation and fractionation will continuously demonstrate their utility and be incorporated in many 3D-LC platforms. Particularly, with the wide-spreading of automated sample handling platforms, these devices will be the future of high throughput sample preparation for 3D-LC. 

## Figures and Tables

**Table 1:** Performance of various offline 3D-LC systems.

3D-LC setup	Sample (Protein Amount)	MS	Identified Proteins *	Identified Unique Peptides	Fraction Number	MS Time (h)	Year, Reference
RP-SCX-RP	Human plasma (500 µg)	Agilent 1100 LC/MSD Trap	484	-	40	100	2006, [57]
SEC-SCX-RP	Human liver (1 mg)	QSTARXL	636	3451	120	206	2007, [55]
SEC-HILIC-RP	Human serum (7.4 mg)	Quadrupole ion trap	1955	-	20	60	2011, [31]
ERLIC- RP- RP	Human serum (1.2 mg)	QTOF	1088	208	144	216	2013, [62]
SCX-RP-RP	Mouse embryonic fibroblast (1 mg)	LTQ Orbitrap XL	5051	29,843	30	-	2013, [58]
SCX-HILIC-RP	K562 cells (3 mg)	LTQ-Orbitrap Velos	4708	22,148	63	126	2013, [67]
SCX-HILIC-RP	HeLa cells (500 µg)	LTQ-Orbitrap Velos	3424	11,980	63	126	2013, [67]
SEC-HILIC-RP	*Paracoccus denitrificans* (8 mg)	Orbitrap Elite	2627	-	36	66	2015, [68]
SCX-RP-RP	Human embryonic stem cell (-)	Thermo Finnigan LTQ	3184	~24,000	100	117	2015, [59]
RP- RP- RP	Jurkat cells (720 µg)	TripleTOF 5600	14,230	251,166	126	189	2016, [61]
GELFrEE-RP-RP & LPIEF-RP-RP	Human urine (5 mg)	TripleTOF 5600	6085	68,151	360	720	2017, [60]
SAX-RP-RP	HEK 293T cells (30 µg)	Orbitrap Fusion	8222	74,432	11	20.4	2017, [69]
SCX & SAX-RP-RP	Human serum (-)	Orbitrap Fusion	862	-	12	12	2018, [70]

* Proteins identified with at least two peptides. A minus sign indicates no reported information.

**Table 2:** Performance of some online 3D-LC systems.

3D-LC Setup	Sample (Amount)	MS	Identified Proteins *	Identified Unique Peptides	Fraction Number	MS Time (h)	Year, Reference
RP-SCX-RP	*Saccharomyces cerevisiae* (200 µg)	LCQ Deca XP	~1900	-	60	140	2005, [76]
RP-SAX-RP	*Saccharomyces cerevisiae* (5 µg)	Orbitrap XL	4004	26,468	101	202	2011, [80]
			3821	25,091	51	102	2011, [80]
RP-SAX-RP	Murine embryonic stem cells (25 µg)	Orbitrap XL	11,352	211,535	20	580	2013, [82]
RP-SCX-RP	PC12 cell (100 µg)	AB Sciex QSTAR XL QTOF	6345	97,309	12	24	2015, [83]

* Proteins identified with at least two peptides.

**Table 3:** Performance of 2 combined offline-online 3D-LC systems.

Pre-Fractionation	LC Setup	Sample (Amount)	MS	Identified Proteins *	Identified Unique Peptides	Fraction Number	MS Time (h)	Year, Reference
RP	SCX-RP	Human liver (200 µg)	LTQ-Orbitrap Velos	2759	-	36	124	2014, [79]
SCX-RP	CZE	Mouse brain (500 µg)	Q-Exactive HF	8200	65,000	60	70	2018, [51]

* Proteins identified with at least two peptides.

**Table 4:** Comparison of online and offline 3D-LC strategies.

Feature	Offline 3D-LC	Online 3D-LC
Setup	Simple	Usually sophisticated
Ability to transfer technologies to other laboratories	High	Low-medium
Sample amount	High, up to several mg Not suitable for samples with a limited amount	Low, from several µg to 100 µg Allow only low-amount samples
Operation	Separated dimensions, simple and flexible to operate	Usually full-automation, well-trained skill required
Sample pooling	Allow	Usually not allow
Sample handling	High	Low
Sample loss	High-medium	Low

**Table 5:** Performance comparison among various 1D, 2D, and 3D-LC systems.

Sample	MS	Paramater	1D	2D	3D	Reference
HEK 293T cells	Orbitrap Fusion	Sample amount (µg)	6	30	30	[69]
		Setup	RP	SAX-RP	SAX-RP-RP	
		Id. proteins	4275 ± 26	5380 ± 88	8222 ± 109	
		Id. unique peptides	19,768 ± 333	34,912 ± 925	74,432 ± 996	
		Fraction number	1	3	11	
		MS time (h)	1.4	4.2	20.4	
Jurkat cells	TripleTOF 5600	Sample amount (µg)	1	200	720	[61]
		Setup	RP	RP-RP	RP-RP-RP	
		Id. proteins	2568	8757	14,230	
		Id. unique peptides	11,878	109,461	251,166	
		Fraction number	1	21	126	
		MS time (h)	1.5	31.5	189	
HeLa cells & K562 cells	LTQ-Orbitrap Velos	Sample amount (µg) *	125 and 750	500 and 3000	500	[67]
		Setup	RP	HILIC-RP	SCX-HILIC-RP	
		Unique phos.pro. *	1570 and 1763	3891 and 4132	3424 and 4708	
		Unique phos.pep. *	3726 and 4104	16,637 and 16,722	11,980 and 22,148	
		Fraction number	1	20	63	
		MS time (h)	2	40	126	
Mouse embryonic fibroblast	AB Sciex QSTAR XL QTOF	Sample amount (µg)		15	15	[83]
		Setup		RP-RP	RP-SCX-RP	
		Id. proteins		2358	3114	
		Id. unique peptides		13,573	25,792	
Human urine	TripleTOF 5600	Sample amount (µg)	20	200	5000	[60]
		Setup	RP	RP-RP	GELFrEE-RP-RP and LPIEF-RP-RP	
		Id. proteins	808	3162	6085	
		Id. unique peptides	13,895	25,940	68,151	
		Fraction number	1	20	360	
		MS time (h)	2 × 3	40	720	
Mouse brain	Q-Exactive HF	Sample amount (µg)		500	500	[51]
		Setup		RP-RP	SCX-RP-CZE	
		Id. proteins		8900	8200	
		Id. unique peptides		70,000	65,000	
		Fraction number		30	60	
		MS time (h)		60	70	
*E. coli*	LCQ Deca XP ion trap	Sample amount (µg)		20	20	[80]
		Setup		RP-RP	RP-SAX-RP	
		Id. proteins		702	923	
		Id. unique peptides		2923	4254	
		Fraction number		40	37	
		MS time (h)		80	74	

Identified proteins are those with at least two peptides. * Two numbers in each position are for HeLa cells and K562 cells, respectively. Id.: Identified; phos.pro.: phosphoproteins; phos.pep.: phosphopeptides.

## Abbreviations

PTMs	Post-translational modifications
MD	Multidimensional
2D	Two-dimensional
3D	Three-dimensional
MudPIT	Multidimensional protein identification technology
SCX	Strong cation exchange
RPLC	Reversed phase liquid chromatography
SAX	Strong anion exchange
SEC	Size exclusion chromatography
HILIC	Hydrophilic interaction chromatography
CZE	Capillary zone electrophoresis
GELFrEE	Gel-eluted liquid fraction entrapment electrophoresis
LPIEF	Liquid-phase isoelectric focusing
ERLIC	Electrostatic repulsion hydrophilic interaction chromatography
ZIC	Zwitterion–ion
StageTips	Stop and go extraction tips
iST	in-StageTip
3D-SISPROT	3D peptide fractionation technology
SILAC	Stable isotope labeling by amino acids in cell culture
TMT	Tandem mass tags
iTRAQ	Isobaric tags for relative and absolute quantitation
DEEP SEQ	DEep Efficient Peptide SEquencing and Quantification
PASEF	Parallel accumulation–serial fragmentation
SWATH	Sequential window acquisition of all theoretical spectra
DIA	Data-independent acquisition
